# Platelets are associated with xenograft tumor growth and the clinical malignancy of ovarian cancer through an angiogenesis-dependent mechanism

**DOI:** 10.3892/mmr.2014.3082

**Published:** 2014-12-11

**Authors:** LEI YUAN, XISHI LIU

**Affiliations:** Department of Gynecology, Obstetrics and Gynecology Hospital, Fudan University, Shanghai 200011, P.R. China

**Keywords:** platelets, xenograft tumor growth, ovarian cancer, angiogenesis-dependent mechanism

## Abstract

Platelets are known to facilitate tumor metastasis and thrombocytosis has been associated with an adverse prognosis in ovarian cancer. However, the role of platelets in primary tumour growth remains to be elucidated. The present study demonstrated that the expression levels of various markers in platelets, endothelial adherence and angiogenesis, including, platelet glycoprotein IIb (CD41), platelet endothelial cell adhesion molecule 1 (CD31), vascular endothelial growth factor (VEGF), lysyl oxidase, focal adhesion kinase and breast cancer anti-estrogen resistance 1, were expressed at higher levels in patients with malignant carcinoma, compared with those with borderline cystadenoma and cystadenoma. In addition, the endothelial markers CD31 and VEGF were found to colocalize with the platelet marker CD41 in the malignant samples. Since mice transplanted with human ovarian cancer cells (SKOV3) demonstrated elevated tumor size and decreased survival rate when treated with thrombin or thrombopoietin (TPO), the platelets appeared to promote primary tumor growth. Depleting platelets using antibodies or by pretreating the cancer cells with hirudin significantly attenuated the transplanted tumor growth. The platelets contributed to late, but not early stages of tumor proliferation, as mice treated with platelet-depleting antibody 1 day prior to and 11 days after tumor transplantation had the same tumor volumes. By contrast, tumor size in the early TPO-injected group was increased significantly compared with the late TPO-injected group. These findings suggested that the interplay between platelets and angiogenesis may contribute to ovarian cancer growth. Therefore, platelets and their associated signaling and adhesive molecules may represent potential therapeutic targets for ovarian cancer.

## Introduction

Ovarian cancer is one of the most life-threatening types of epithelial cancer and patients often experience rapid relapse following chemotherapy. Abnormal thrombosis and accompanied thrombocytosis can occur in ovarian cancer patients and it has been suggested that these two factors are involved in cancer metastasis. Platelets promote metastasis by protecting tumor cells from destruction by immune cells (1). They also enhance tumor cell adhesion through a selectin-GPIIb-dependent mechanism. Deficiencies in the expression of P-selectin by platelets may prevent cancer cell rolling and their adhesion to endothelial cells under flow conditions, thereby reducing the number of metastatic lesions (2). A previous study suggested that platelets mediate the epithelial-mesenchymal transition (EMT) of cancer cells (3), as platelet-derived transforming growth factor-β (TGFβ) and direct platelet-tumor cell contact activated the TGFβ/Smad and NF-κB pathways. This induced the transition of tumor cells into an invasive mesenchymal-like phenotype and enhanced tumor metastasis *in vivo*.

Although the role of platelets in cancer migration is well understood, the details of how platelets are involved in cancer proliferation remain to be elucidated. There is evidence indicating that platelets cause cell cycle arrest in cancer cells, however, thrombocytosis occurs in 10–50% of patients with solid malignancies (4). This has been labeled as ‘reactive’ or ‘secondary’, implying that the tumor, even small tumors, can stimulate platelet production (5). In addition, thromboipoetin (TPO), which drives thrombopoiesis, is often used to regenerate the platelet counts in patients following chemotherapy, a treatment that has been associated with tumor homing (6). Therefore, further clarification of the association between platelets and tumor proliferation may assist in elucidating the mechanisms underlying carcinogenesis and is of clinical importance (7).

In the present study, the correlation between thrombogenic markers and malignancies of ovarian tumor was investigated. The expression of coagulant factors from patients with benign, borderline and malignant phenotypes were determined and human tumor tissue samples were stained using antibodies against platelet glycoprotein IIb (CD41), a marker for platelets, and other biomarkers associated with angiogenesis, including platelet endothelial cell adhesion molecule 1 (CD31), vascular endothelial growth factor (VEGF), lisyl oxidase (LOX), focal adhesion kinase (FAK) and breast cancer anti-estrogen resistance 1 (BCAR1). The roles of platelets in ovarian cancer growth were also determined in a transplanted mouse model. Tumor growth patterns, animal survival rate, platelet-depletion and infusion in the murine tumor model were assessed. The results provided evidence that platelets contribute to the proliferation of ovarian cancer by mediating angiogenesis, which may be important for the development of intervention strategies to treat ovarian cancer.

## Materials and methods

### Patients and specimens

A total of 140 patients with histologically confirmed ovarian tumors, including serous cystadenoma (SC), borderline serous cystadenoma (BSC), serous cystadenocarcinoma (SC), mucinous cystadenoma (MC), borderline mucinous cystadenoma (BMC), mucinous cystadenocarcinoma (MCC) and clear cell carcinoma (CCC), were recruited by the Obstetrics and Gynecology Hospital of Fudan University between 2008 and 2010. Each histotype contained 20 tissue specimens. The patients all underwent primary surgery at the the Obstetrics and Gynecology Hospital of Fudan University for pelvic masses. Surgery was advised if the diameter of the pelvic mass exceeded 5 cm, and patients with physioloigal ovarian cysts were excluded. International Federation of Gynecological Oncologists (FIGO) staging was assigned to the tumors following surgery if possible.

The patient charts were reviewed to obtain clinicopathological data regarding age, preoperative blood coagulation function (activated partial thromboplastin time, prothrombin time, international normalized ratio, fasting blood glucose and D-dimer), pre- and post-operative platelet level, preoperative serum levels of CA125, CA199, carcinoembryonic antigen and α-fetoprotein, diagnosis, histology, FIGO staging (if any), residual disease following tumor cytoreductive surgery (if any) and overall survival rate. Tissue blocks, which were harvested at the time of surgery and subsequently formalin fixed, paraffin embedded and archived in the pathology department of the hospital in the dark at room temperature, were retrieved for further immunohistochemical (IHC) staining.

Additional fresh tissue specimens from 12 patients (six with malignant epithelial ovarian tumors and six with benign tumors) were collected following patient consent for immunofluorescent staining.

The present study was reviewed and approved by the Ethics Committee of the Obstetrics and Gynecology Hospital of Fudan University, Shanghai, China.

### IHC

The tissue samples, obtained from all 140 patients with benign, borderline and malignant ovarian tumors, were retrieved and serial 4-mm sections were obtained from each block. The first slide was stained for hematoxylin and eosin to confirm the pathological diagnosis and the subsequent slides were stained for CD31, CD 41, FAK, LOX, BCAR1 and VEGF, respectively. Routine deparaffinization and rehydration procedures were performed following online protocols (http://www.bioworldantibodies.com/info/IHC.pdf).

For antigen retrieval, the slides were heated at 98°C in either an EDTA-Tris buffer (pH 9.0) or citric acid for 30 min and cooled naturally to room temperature. EDTA-Tris buffer was used for the FAK and LOX staining, while citric acid was used for the CD31, CD41, BCAR1 and VEGF staining. Endogenous peroxidase was blocked using 3% H_2_O_2_ in methanol for 10 min prior to washing with phosphate-buffered saline (PBS). The sections were then incubated with the following primary antibodies: rabbit anti-CD31 polyclonal antibody (1:50; ab28364), rabbit anti-CD41 polyclonal antibody (1:200; ab63983), rabbit anti-FAK polyclonal antibody (1:200; ab4803), rabbit anti-LOX polyclonal antibody (1:200; ab31238), rabbit anti-BCAR1 polyclonal antibody (1:150; ab31831) and rabbit anti-VEGF polyclonal antibody (1:200; ab46154) (Abcam, Cambridge, UK) overnight at 4°C. The secondary antibody used in this study was Sunpoly-H II (Shanghai Sunbio Co., Shanghai, China). The slides were rinsed and incubated with horseradish peroxidase-labeled secondary anti-rabbit antibody detection reagent (Shanghai BioTech Company, Ltd., Shanghai, China) at room temperature for 30 min. The bound antibody complexes were stained for 3–5 min, or until appropriate for microscopic examination with diaminobenzidine and then counterstained with hematoxylin (30 sec) and mounted. Negative controls were generated by incubating slides with PBS instead of the primary antibody.

The immunoreactivity staining was characterized quantitatively using a semi-quantitative scoring system, as described previously (8). The number and intensity of positive cells was quantified using Image-Pro Plus 6.0 software (Media Cybernetics, Inc., Rockville MD, USA) in a blinded-manner. A series of five random images on several sections were captured to obtain a mean value.

The microvascular density (MVD) of the containing CD31-stained slides was assessed using light microscopy (Eclipse Ni-U; Nikon Corp., Tokyo, Japan) in the areas having the highest number of capillaries and small venules (neovascular hot spots). Subsequently, the microvessels were counted on 10 selected (x200) fields of the ‘hot plot’ by the same investigator in a blinded-manner. The MVD was defined as the mean vessel count obtained in these fields, as reported by Ma *et al* (9).

### Immunofluorescent staining

The freshly harvested tissues were formaldehyde-fixed prior to freezing and were sectioned using a cryostat (Leika CM1850; Leica Microsystems GmbH, Nussloch, Germany) to 4 μm. The tissue sections were then incubated with rabbit anti-CD41 polyclonal antibody (1:100; ab63983), mouse anti-CD31 monoclonal antibody (1:100; ab24590) and/or mouse anti-VEGF monoclonal antibody (1:200; ab1316), which were all purchased from Abcam, overnight at 4°C. The tissue sections were then incubated with a secondary goat anti-rabbit fluorescein isothiocyanate (111-095-003; Jackson ImmunoResearch Laboratories, Inc., West Grove, PA, USA) and goat anti-mouse Rhodamine (115-295-003; Jackson ImmunoResearch Laboratories, Inc.) at 37°C for 1 h. The nuclei were counterstained with diamidinophenylindole (Sigma-Aldrich, St. Louis, MO, USA) and fluorescent images were captured using microscopy (BX51; Olympus, Tokyo, Japan) and analyzed using Image-Pro Plus software for colocalization.

### Tumor growth and survival rates in the mouse model

To assess the effects of different treatments on the tumor growth and mouse survival rates, SKOV3 human ovarian cancer xenografts in athymic nude mice were used. The animals were purchased from Biomodel (Shanghai Research Center for Model Organisms), and were kept under standard conditions of 25°C, 40–60% humidity and a 12-h light/dark cycle, and access water and food *ad libitum*. Tumor loci were generated by injecting 4×10^6^ tumor cells subcutaneously into 6–8-week-old female mice (weight, 18–20 g) on day 0.

For the thrombin and hirudin treatment groups, prior to tumor transplantation, the ovarian tumor cells were pretreated with thrombin, an activator of platelets (1 U/10^6^ cells; Sigma-Aldrich) or hirudin, a specific inhibitor of thrombin (0.1 μg/10^6^ cells) donated by Professor Houyan Song, Fudan University (Shanghai, China) for 10 mins. The other groups were treated 7 days after tumor transplantation with either: Platelet depletion, involving administration of rat anti-mouse GPIb immunoglobulin (Ig)G antibody (2 μg/g, Emfret Analytics, Eibelstadt, Germany) via the tail veins once every 5 days; Isotype control, involving administration of rat IgG (2 μg/g, Emfret Analytics) via the tail veins once every 5 days; platelet infusion, in which the platelets first separated from the platelet-rich plasma of the blood by centrifugation, washed by gel filtration and resuspended in buffer containing 137 mm NaCl, 4 mm KCl, 0.5 mm MgCl_2_, 0.5 mm Na_2_HPO_4_, 11.1 mm dextrose, 0.1% bovine serum albumin and 10 mm *N*-2-hydroxyethylpiperazine-*N*′-2-ethanesulfonic acid (pH 7.4), as previously described (10). Briefly, in the platelet infusion group, platelets were first separated from the platelet rich plasma (PRP) of blood by centrifugation at 280 × g for 8 min, the plasma and buffy coat were gently transferred to a fresh tube and the centrifugation was repeated at 280 × g for 4 min. Platelets were isolated by filtering the resulting PRP through a Sepharose 2B (Sigma-Aldrich) column equilibrated with Pipes buffer (25 mM Pipes, 137 mM NaCl, 4 mM KCl, 0.1% dextrose; pH 7.0) and then resuspended in buffer containing 137 mM NaCl, 4 mM KCl, 0.5 mM MgCl_2_, 0.5mM Na_2_HPO_4_, 11.1 mM dextrose, 0.1% bovine serum albumin, 10mM *N*-2-hydroxyethylpiperazine-*N*′-2-ethanesulfonic acid (pH 7.4). The platelets (6×10^8^) from two donor mice in buffer were then injected into a single recipient xenograft mouse via the tail vein and platelet infusion was repeated every 5 days until experimental termination or TPO, involving administration of recombinant human (rh)TPO (50 U/mouse/day; 3Sbio, Inc., Shenyang, China) for 7 days.

The tumor volume and the number of mice remaining alive following tumor cell xenograft (lethalities were due to dyscrasia of the tumor growth) were measured on days 7, 12, 17, 22, 27, 32 and 37. The volumes of the *in situ* tumors were defined as V=πab^2^/6 (a, major diameter; b, minor diameter) (11) and measured using calipers.

### Effect of platelets on early developing and fast growing periods in vivo

Based on previous *in vitro* studies demonstrating the effects of platelets on the promotion of primary ovarian tumor growth (12), the present study evaluated the role of platelets in tumor growth during different growing periods of carcinogenesis (early developing, vs. fast growing periods).

In the early platelet depletion group (E-PD/E-non-immune), the mice received either rat anti-mouse GPIb or control rat IgG antibodies via the tail vein 1 day prior to subcutaneous inoculation of the tumors (−1 day). The antibody regimes were repeated every 5 days until experimental termination. In the late platelet depletion group (L-PD/L-non-immune), the mice received either rat anti-mouse GPIb or control rat IgG via the tail vein 11 days post-tumor inoculation, which was repeated every 5 days until experiment termination.

In the early TPO treatment group (E-TPO), from -4 day, the mice were injected subcutaneously with rhTPO for 4 days. The mice then received injection once every 4 days (rhTPO injection on days 1 and 5). For the late-TPO treatment group (L-TPO), starting from day 11, the mice received with TPO for 4 days (between days 11 and 14). The purpose of starting the TPO injections at -4 days for the E-TPO group was to enable the platelet counts to rise 2-fold above that of the control at day 0. This treatment enabled the platelet count to be comparable to that of platelet infusion.

Each of the E-TPO, E-PD, E-non-immune, L-TPO, L-PD and L-non-immune groups were comprised of 8 mice. For all treatment groups, the mice were sacrificed by cervial dislocation on day 21. The volumes of the *in situ* tumors were defined as *V=*πab^2^/6, measured using calipers.

### Statistical analysis

For descriptive statistics, box plots were used to graphically depict groups of immunoreactivity data and clinical parameters. The bottom and top of the box represent the lower and upper quartile, respectively, the band close to the middle of the box represents the median and open circles indicate outliers (values >1.5 × interquartile range). Pearson’s or Spearman’s rank correlation coefficient were used to evaluate the correlations between the variables when the two variables were continuous or when at least one variable was ordinal. The association between various clinical and pathological parameters was compared using χ^2^ tests. The present study compared continuous variables using Student’s t-test or analysis of variance (ANOVA) and used non-parametric tests (Mann-Whitney), when appropriate, to compare differences. P<0.05 was considered to indicate a statistically significant difference. All statistical analyses were performed using SPSS 16.0 software (SPSS, Inc., Chicago, IL, USA).

## Results

### Patients with a poor prognosis are prone to thrombophilia

**Figure 1:**
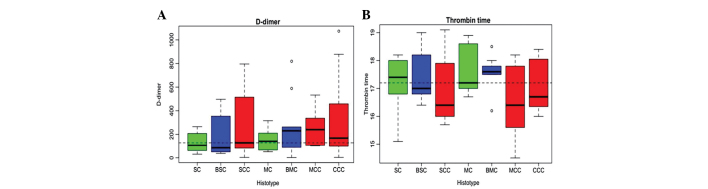
(A) Malignant histotypes (SCC, MC and CCC) had higher levels of D-dimer (marker for fibrin generation) and (B) shorter thrombin clotting time scompared with the benign samples. The coagulation indices of clinical specimens from 140 patients with SC, BSC, SCC, MC, BMC, MCC, CCC were analyzed. No differences in ages were observed among clinical categories (mean=46.1, standard deviation=17.8). Bars represent the maximum and minimum values, open circle indicate outliers. SC, serous cystadenoma; BSC, borderline serous cystadenoma; SCC, serous cystadenocarcinoma; MC, mucinous cystadenoma; BMC, borderline mucinous cystadenoma; MCC, mucinous cystadenocarcinoma; CCC, clear cell carcinoma.

To compare the differential expression levels of coagulation factors and molecular markers among a variety of human pathological conditions, clinical specimens from 140 patients with SC, BSC, SCC, MC, BMC, MCC and CCC were used for analysis of coagulation indices and IHC markers (each histotype had 20 specimens, respectively). No differences in ages were apparent among the clinical categories (mean=46.1; standard deviation=17.8). The malignant histotypes (SCC, MC and CCC) had a higher D-dimer level, a marker for fibrin generation (Fig. 1A) and a shorter thrombin clotting time compared with the benign samples, as analyzed by ANOVA (P<0.05; Fig. 1B). Prior to surgery, the carcinoma patients had a significantly higher platelet count compared with either the borderline cystadenoma or cystadenoma (281±103×10^9^, 262±108×10^9^, 209±41×10^9^, respectively; P=0.03). By contrast, following tumor removal surgery, the platelet counts were equal in the three groups. Notably, the preoperative platelet counts were positively correlated with the clinical stages in the malignant histotypes (r=0.494; P=0.004).

**Figure 2:**
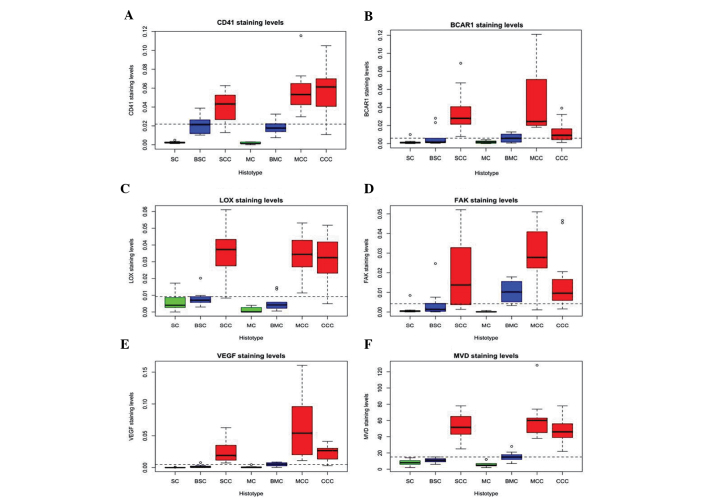
Differences in the expression levels of CD41, BCAR1, LOX, FAK, VEGF and CD31 (MVD) in different histoypes. (A) CD41; (B) BCAR1; (C) LOX; (D) FAK; (E VEGF and (F) MVD staining among the different histotype groups. Clinical specimens from 140 patients with SC, BSC, SCC, MC, BMC, MCC and CCC were subject to analysis of immunohistochemical markers. No differences in ages were observed among the clinical categories (mean=46.1, standard deviation=17.8). Bars represent the minimum and maximum values, open circles indicate outliers. SC, serous cystadenoma; BSC, borderline serous cystadenoma; SCC, serous cystadenocarcinoma; MC, mucinous cystadenoma; BMC, borderline mucinous cystadenoma; MCC, mucinous cystadenocarcinoma; CCC, clear cell carcinoma; LOX, lysyl oxidase; FAK, focal adhesion kinase; VEGF, vascular endothelial growth factor; MVD, microvascular density.

**Figure 3:**
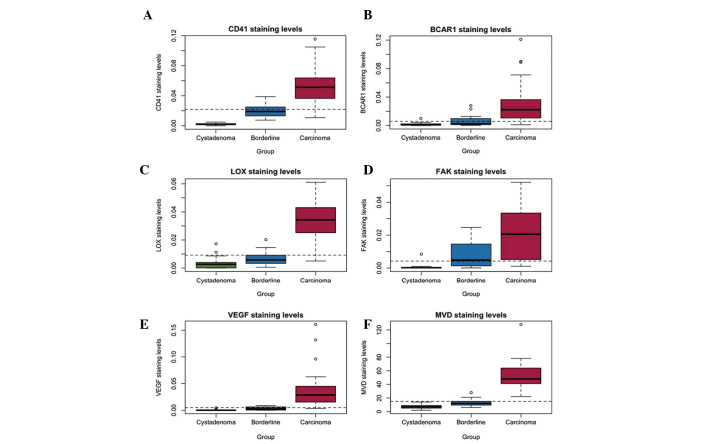
Expression levels of CD41, BCAR1, LOX, FAK, VEGF and MVD in patients with carcinoma, borderline cystadenoma or cystadenoma group. (A) CD41, (B) BCAR1, (C) LOX, (D) FAK, (E) VEGF and (F) MVD staining among the different histotype groups. Histotypes were regrouped into cystadenoma, borderline and carcinoma. Bars represent the minimum and maximum values, open circles indicate outliers. CD41, platelet glycoprotein IIb; BCAR1, breast cancer anti-estrogen resistance 1; LOX, lysyl oxidase; FAK, focal adhesion kinase; VEGF, vascular endothelial growth factor; MVD, microvascular density.

To analyze the phenotypes of the cancer specimens, angiogenic, tumorigenic and thrombogenic markers, including CD41, CD31 (MVD), BCAR1, VEGF, LOX and FAK, were specifically selected. CD41, a platelet marker and receptor (GPIIb/IIa), mediates platelet adhesion to endothelial cells and augments endothelial angiogenesis (13). Tumor cell adhesion to endothelium-bound platelets is mediated by GPIIb-IIIa in flow (14). BCAR1, also referred to as p130 Crk-associated substrate, is located at the sites of adhesion and is associated with tumor migration, colony formation and anchorage-independent growth (15–18). FAK is responsible for the uninhibited proliferation of cancer cells, their protection from apoptosis, invasion, migration, adhesion and spreading, as well as tumor angiogenesis (19). VEGF also induces angiogenesis and tumor growth (20). There were significant differences in the expression levels of CD41, BCAR1, LOX, FAK, VEGF and CD31 (MVD) in the different histoypes (P<0.05; Fig. 2A–F). Regrouping of the seven histotypes into three groups: cystadenoma, borderline cystadenoma and carcinoma, according to their outcomes, revealed that patients with carcinoma expressed significantly more CD41, BCAR1, LOX, FAK, VEGF and MVD compared with the borderline or cystadenoma groups (Fig. 3A–F). In addition, the expression levels of CD41, CD31 (MVD), BCAR1, VEGF, LOX and FAK were positively correlated with the clinical stages in the carcinoma (SCC, MC and CCC) samples (r=0.528, 0.613, 0.575, 0.575, 0.43, 0.571 and 0.589, respectively). The expression levels were significantly higher in patients diagnosed with late stage cancer compared to those with early stage cancer (P<0.05). Notably, the serum CA-125 levels were positively correlated with the expression levels of CD41 and MVD (data not shown).

**Figure 4:**
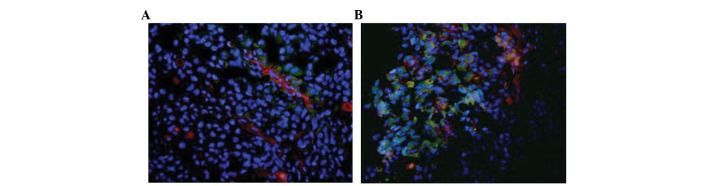
Colocalization of the platelet marker CD41 (green) with (A) CD31 (red) or (B) VEGF (red) in human ovarian mucinous cystadenocarcinoma. Tissues (4μm) were incubated with anti-CD41, CD31 and VEFG antibodies prior to staining with fluorescein isothiocyanate and rhodamine-conjugated secondary antibodies. The nuclei were stained using diamidinophenylindole (blue). The samples were analyzed by fluorescence microscopy (magnification, 400x). CD41, platelet glycoprotein IIb;;CD31, platelet endothelial cell adhesion molecule 1; VEGF, vascular endothelial growth factor.

In the carcinoma samples, the expression of CD41 overlapped with that of CD31 and VEGF (Fig. 4A and B), suggesting that the platelets may contribute to the generation of carcinoma by inducing angiogenesis. Furthermore, platelets formed aggregates surrounding the tumor.

### Effects of platelets and thrombosis on cancer and survival rates in a xenograft mouse model

**Figure 5:**
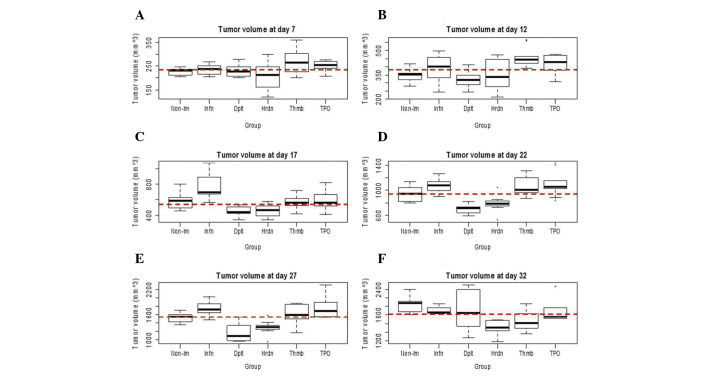
Effects of platelets in growth of tumor volume. Nude mice were subcutaneously inoculated with SKOV3 tumor cells and grouped according to treatment type: Non-immune group (injection of platelet non-immune antibody with 2 μg/g control rat IgG every 5 days); depletion group (injecting 2 μg/g platelet depletion rat anti-mouse GPIb IgG antibody every 5 days); infusion group (infusing platelets from two donor mice into a single recipient every 5 days); TPO group (subcutaneous injection of 50 U/mouse/day TPO for 7 days); Thrombin group (thrombin treatment prior to subcutaneous transplantation); hirudin group (hirudin treatment prior to subcutaneous transplantation) and control group (tumor subcutaneous transplantation without any treatment). The tumor volumes (mm^3^) were recorded on days (A) 7, (B) 12, (C) 17, (D) 22, (E) 27 and (F) 32. Bars represent the minimum and maximum values, open circles indicate outliers. Non-Im, non-immune group; Infn, infusion group; Dplt, depletion group; Hrdn, hirudin group; Thmb, thrombin group; TPO, thrombopoietin.

During several stages of tumor development, tumor cells acquire different capacities to sustain proliferation, evade growth suppressors and activate invasion (21). The interactions between tumors and platelets in blood vessels are critical for tumor attachment and transendothelial migration. To examine the role of platelets and coagulation in tumor development *in situ*, nude mice were subcutaneously transplanted with SKOV3 ovarian tumor cells prior to further treatments, including platelet depletion, platelet infusion, TPO injection and thrombin and hirudin pretreatment. The depletion of platelets with the injection of anti-GPIb IgG resulted in a decrease in tumor volume compared with the injection of control non-immune antibody alone (Fig. 5). The inhibition of tumour growth by platelet depletion remained after 5 days (Fig. 5B). In addition, the effect of platelet depletion was increasingly potent with tumor progression (Fig. 5B–E). At day 27, platelet depletion reduced tumor volume by 29% compared with the control samples (Fig. 5E). However, the mice receiving platelet infusion exhibited a 1.14-fold increase in their median tumor size at day 12. The tumor-promoting effect of platelets was sustained until day 27. Notably, at day 32 the median tumor size of the platelet infusion group was comparable to that of the control (Fig. 5F), which was possibly due to the mice succumbing to mortality.

The injection of TPO, which is a factor regulating platelet production, resulted in the most profound induction of tumor growth day 12 and between days 22 and 27 (Fig. 5B–E). When the transplanted tumor was pre-incubated with hirudin, an antithrombin reagent, the tumor proliferation was reduced (Fig. 5A–E). Furthermore, the tumor-suppression effect of hirudin peaked at day 32. However, the tumor-supressing effect of hirudin treatment was less significant than that of platelet depletion. Pretreatment of the tumor cells with thrombin, an enzyme that converts fibrinogen to fibrin, increased the tumor size compared with the non-immune antibody control (Fig. 5A–D). The difference was observed from the beginning of tumor measurement until day 27.

**Figure 6:**
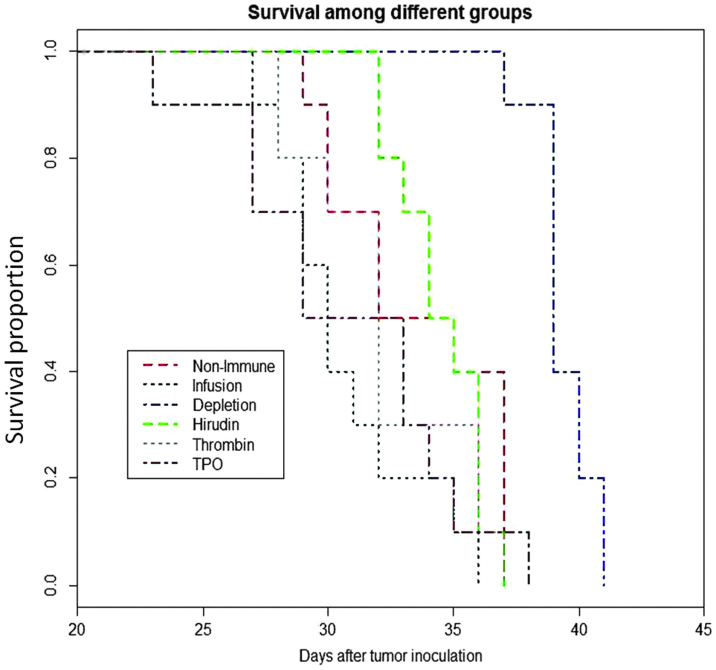
Effect of platelets on mouse survival rates. Nude mice were subcutaneously inoculated with SKOV3 tumor cells and grouped according to different treatments. Non-immune group (injection of platelet non-immune antibody with 2 μg/g control rat IgG every 5 days); depletion group (injecting 2 μg/g platelet depletion rat anti-mouse GPIb IgG antibody every 5 days); infusion group (infusing platelets from two donor mice into a single recipient every 5 days); TPO group (subcutaneous injection of 50 U/mouse/day TPO for 7 days); Thrombin group (thrombin treatment prior to subcutaneous transplantation); hirudin group (hirudin treatment prior to subcutaneous transplantation) and control group (tumor subcutaneous transplantation without any treatment). The proportion of mice remaining alive were recorded on days 7, 12, 17, 22, 27, 32 and 37. TPO, thrombopoetin; Ig, immunoglobulin

The effect of different treatments on the survival rate of mice bearing a human ovarian tumor are shown in Fig. 6. The median survival rate for the non-immune antibody treated group was 33.5 days. Exogenous infusion of platelets resulted in regional transplant tumor growth and reduced the median survival rate to 30 days (Fig. 6). The median survival rate for mice receiving platelet-depleting antibody, leading to experimental thrombocytopenia, was 39 days. In addition, 9 of the 10 mice receiving the platelet-depleting antibody survived >39 days.

Pretreatment of the tumor cells with hirudin increased the mouse survival rates. By contrast, pretreatment with thrombin decreased the mouse survival rates. Notably, TPO had a dual effect on survival rate. Initially, it caused a rapid decrease in survival rate, while at the later time point, it prolonged the rates. TPO has been suggested to have additional functions to promoting platelet generation, which may affect survival rates in a non-specific manner (22).

### Effects of platelets at different ovarian cancer developmental stages

**Figure 7:**
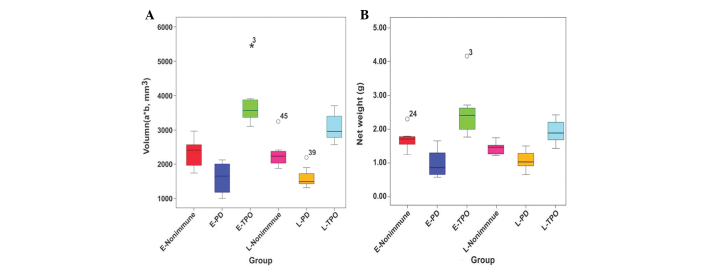
Effects of E-PD and L-PD (rat antimouse GPIb IgG antibody, 2 μg/g): E-non-immune and L-non-immune (control rat IgG, 2 μg/g) and E-TPO and L-TPO on (A) tumor volumes (πab2/6, mm^3^) and (B) net tumor weight (g). n=8 in each group. Bars represent the minimum and maximum values, open circles indicate outliers. Nonimmune, non-immune antibody injection; PD, platelet-depletion; TPO, thrombopoetin, E-, early; L-, late; Ig, immunoglobulin.

To assess the roles of platelets at different cancer developmental stages (early developing and fast growing periods), two different methods of platelet depletion and TPO injection were developed, as mentioned previously. E-TPO treatment led to a significantly larger tumor volume (3778.1±737.7 mm^3^) and stripped tumor weight (2.48 g) compared with L-TPO treatment (3,075.9±405.9 mm^3^ and 1.91 g) as shown in Fig. 7A and B (P<0.05). By contrast, E-PD treatment resulted in smaller tumor volume (1,599.0±435.9 mm^3^) and stripped tumor weight (0.9 g) compared with those treated with L-PD (1,660.8±294.1 mm^3^ and 1.06 g; Fig. 7A and B), although the difference was not statistically significant (P>0.05). For the control groups, tumor volume (2,327.9±410.2 mm^3^) and stripped tumor weight (1.69 g) following E-non-immune treatment were not significantly different compared with those of the L-non-immune treatment group (2,301.5±423.7 mm^3^ and 1.42 g; Fig. 7A and B).

**Figure 8:**
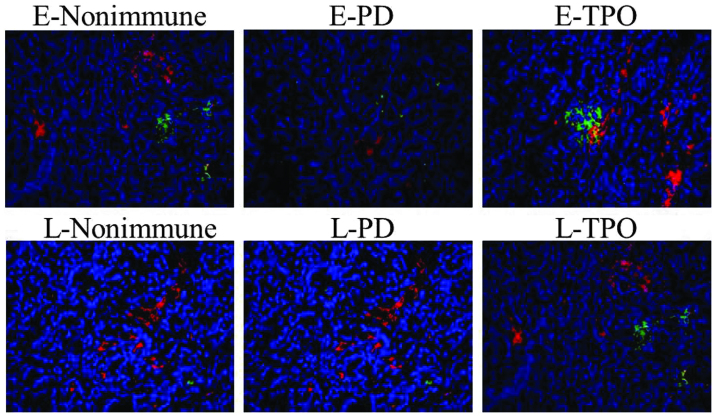
Effects of E-PD and L-PD and E-TPO and L-TPO treatment on the colocalization of CD41 and CD31 in tumor tissues. Images were captured under ×400 magnification using a fluorescent microscope. Blue, diamidinophenylindole; green, CD41 and red, CD31. Nonimmune, non-immune antibody injection; PD, platelet-depletion; TPO, thrombopoietin; E-, early; L-, late; CD41, platelet glycoprotein IIb CD31, platelet endothelial cell adhesion molecule 1.

Fluorescent staining revealed that TPO treatment resulted in more significant overlapping of CD41 and CD31 compared with non-immune antibody treatment (Fig. 8). Mice in the E-TPO group had a higher levels of CD41 and CD31 colocalization compared with those in the L-TPO group.

## Discussion

Thrombocytosis is a notable characteristic associated with cancer progression. However, despite the fact that the metastasis-promoting effect of platelets has been investigated, the role of platelets in primary tumor proliferation remains to be full elucidated. A number of studies have suggested that platelets facilitate hematogenous metastasis (1,2,14,23–25). In blood flow, tumor cells roll and firmly adhere to endothelium-bound platelets. This dynamic adhesion process is dependent on P-selectin and glycoprotein IIb (2,14,25). Deficiency in these two proteins reduces the number of metastatic lesions (2). The present study demonstrated that tumour malignancy was positively correlated with the enhanced expression of CD41, CD31 (MVD), BCAR1, FAK, LOX and VEGF using IHC. By examining human ovarian cancer cells transplanted into nude mice, the present study demonstrated that platelets were relevant for tumor development and mouse survival rates. Early treatment with TPO resulted in a larger tumor loci compared with later treatment, suggesting that efficient neoplasia requires angiogenesis and platelets.

The clinical samples derived from malignant carcinoma had higher expression levels of thrombogenic and angiogenic markers, including D-dimer, based on IHC analysis. D-dimer is a degraded product of fibrin generation (26,27) and its presence, which reflects the activation of the coagulation system and fibrinolysis, this has been associated with clinical tumor growth, invasion and metastasis (27). The present study revealed that malignant cancer patients had a shorter thrombin time, suggesting that the production of thrombin increased. In the tumor microenvironment, thrombin can bind to its receptor PAR-1 on the endothelium and tumor cells, inducing angiogenesis and cancer proliferation (28). Certain types of cancer, including colon carcinoma and melanoma, express tissue factors on their surface to augment local thrombogenesis. However, whether increased coagulation is the cause or the consequence of malignancy of cancer requires further investigation.

BCAR1 is one of the Crk-associated substrate protein family members and a variety of mutations in this protein confers susceptibility to ovarian cancer (16). Reduction in the expression levels of BCAR1 resulted in reduced tumor growth following docetaxel chemotherapy. BCAR1 forms a phosphorylation-dependent signaling complex with FAK and Src kinase, promoting the adhesion-mediated breast cancer survival rate (29). FAK is an important adhesion regulator, which has been suggested to promote cancer metastasis, angiogenesis and tumor progression (30). Elevated levels of FAK in serous ovarian carcinoma are associated with decreased patient survival rates (31). Studies have also suggested that VEGF-induced vascular endothelium permeability is mediated by FAK (32). The present study revealed that malignant cancer patients had increased levels of FAK, BCAR1 and VEGF. These findings implied that increased ‘cross-talk’ of platelets with either soluble factors or P-selectin may enhance tumor survival rate.

The expression levels of the LOX family proteins, which are secreted by tumors and have been investigated extensively, increased in various types of cancer including head and neck squamous cell carcinoma (33), breast (33,34), colorectal (35,36) and prostate (37) cancer. Although numerous studies have supported the roles of the LOX family members in tumor suppression and in promoting metastasis, a number failed to address the underlying mechanism. The potential contributions of LOX, BCAR1 and FAK in the malignant progression of ovarian cancer requires further investigation.

The observation that the expression pattern of VEGF overlapped with that of CD41, suggested that platelet aggregation may promote angiogenesis and tumor proliferation. The coexpression of CD41/CD31 and CD41/VEGF in tissue samples implied that platelets promoted local ovarian cancer growth through a mechanism of pro-angiogenesis. The effect of platelets on angiogenesis may be dependent on the secretion of endothelial growth factors, including platelet-derived growth factor (38). Further investigation using a platelet-tumor co-culture model are required to delineate the soluble factor expression levels.

Thrombocytosis was observed in patients with carcinomas. The cause of malignancy-associated thrombocytosis has been assumed to be attributable to interleukin-6 (IL-6) which potently promotes megakaryocyte maturation and enhances platelet production, although the cellular source of IL-6 may differ between patients (39). Using a murine tumor model, the present study demonstrated that platelet depletion or pretreatment with hirudin considerably reduced the tumor volume and increased the median mouse survival rate. These results suggested that platelets were important for tumor growth. In addition, the mice with early platelet-depletion occurred at an early stage, had a smaller tumor size compared with mice with late stage platelet-depletion. Furthermore, mice receiving early TPO injection had a larger tumor size compared with those receiving late TPO injection. However, whether the produced platelets affected tumor growth and angiogenesis remains to be elucidated. Levels of IL-6 are frequently increased in tumor patients and increases thrombopoiesis only in the presence of TPO, which drives thrombopoiesis. These findings are supported by the demonstration *in vivo* that platelets transfused into mice with orthotopic ovarian tumors enhanced the proliferation index of the tumors. TPO regulates all stages of platelet production by promoting the proliferation and maturation of megakaryocyte progenitors (22). In preclinical studies, TPO treatment resulted in a rapid increase in platelet counts, but not cytokines (40,41). Truncated or full-length forms of TPO can stimulate the production of megakaryocytes and platelets in humans and enhances platelet recovery following chemotherapy and TPO may offer potential in treating thrombocytopenia in cancer patients. In the present study, TPO was administered into the tumor xenograft mice, resulting in ovarian cancer proliferation and reduced survival rates. Therefore, clinically, administration of TPO into patients with the intention of recovering platelet counts following chemotherapy may facilitate latent tumor growth and promote relapse.

Rupture of atherosclerotic plaques exposes the subendothelial collage, which then activates platelets. Activated platelets express receptors, including GPIb-IX, which is the receptor for von Willebrand factor and induces platelet aggregation (42). Platelet aggregation also requires the binding of integrin GPIIB-IIIa receptor to fibrinogen. Therefore, platelets may function as a bridge, which assists in the attachment to blood vessel walls. It has also been demonstrated that platelets could enhance the proliferation rate of human and murine ovarian cancer cells by a mechanism that requires direct cell contact (43). These findings were supported in the present study *in vivo,* in which platelet transfusions into mice with orthotopic ovarian tumors enhanced the tumour proliferation index.

The present study compared the effect on tumor growth of early and late platelet depletion by TPO treatment. The purpose of starting E-TPO from -4 day was to raise platelet counts by 2-fold on day 0. The results indicated that tumour volume when platelet depletion occurred at an early stage of tumor formation (−1 day), were not significantly different compared with platelet depletion at an late stage of tumor formation (11 days). If platelet counts were increased by TPO, the tumor volume in early treatment samples were significantly larger compared with these in late treatment. This implied that platelets did not contribute to the early stages of tumor development, however, when tumors reached a certain size, the role of platelets in tumor proliferation was manifested. As platelets may secrete signaling/adhesive molecules, including TGF, which promote angiogenesis and initiate tumor EMT, they possibly contributed to the later stage of cancer development.

In conclusion, the present study provided *in vivo* evidence that platelets enhanced cell proliferation in ovarian cancer. Additionally, the clinical data suggested that malignant carcinoma cells were characteristic of platelet, angiogenesis and migratory markers. These findings suggested that thrombosis and angiogenic processes may be associated with tumor progression. The results of the present study assist in understanding the underlying mechanism of ovarian cancer growth and provide insight into new therapeutic targets for treating ovarian cancer.

## Abbreviations

CD31: platelet endothelial cell adhesion molecule 1
CD41: platelet glycoprotein IIb
TPO: thrombopoietin
IHC: immunohistochemistry
FAK: focal adhesion kinase
LOX: lysyl oxidase
BCAR1: breast cancer anti-estrogen resistance 1
VEGF: vascular endothelial growth factor
SC: serous cystadenoma
BSC: borderline serous cystadenoma
SCC: serous cystadenocarcinoma
MC: mucinous cystadenoma
BMC: borderline mucinous cystadenoma
MCC: mucinous cystadenocarcinoma
CCC: clear cell carcinoma
MVD: microvascular density
FIGO: international federation of gynecology and obstetrics
EMT: epithelial-mesenchymal transition
